# Longitudinal Plasma Lipidomic Changes in Chinese Patients with Nonvalvular Atrial Fibrillation During 12 Weeks of Edoxaban Treatment

**DOI:** 10.3390/metabo16090672

**Published:** 2026-09-11

**Authors:** Cheng Cui, Junhao Chen, Haodi Chai, Dongyang Liu

**Affiliations:** 1 Drug Clinical Trial Center, Department of Pharmacy, Peking University Third Hospital, Beijing 100191, China; 2 School of Pharmaceutical Sciences, Capital Medical University, Beijing 100069, China

**Keywords:** nonvalvular atrial fibrillation (NVAF), edoxaban, lipidomics, longitudinal study, time-point discrimination

## Abstract

**Introduction:** Altered lipid metabolism is implicated in atrial fibrillation, but longitudinal lipidomic changes during anticoagulation remain poorly characterized. **Objectives:** To characterize plasma lipidomic changes during 12 weeks of edoxaban treatment and explore associated metabolic pathways in Chinese patients with nonvalvular atrial fibrillation (NVAF). **Methods:** This prespecified substudy of a prospective multicenter study analyzed fasting predose plasma collected at baseline and week 12 from 74 patients receiving protocol-assigned edoxaban doses (15 mg, *n* = 3; 30 mg, *n* = 23; 60 mg, *n* = 48). Widely targeted UPLC-MS/MS lipid profiling, with laboratory quality control (QC CV < 30%), yielded 1099 lipid features retained for statistical analysis. Differential lipids were defined by paired testing with Benjamini–Hochberg correction (|log_2_FC| > 0.58 and q < 0.05). **Results:** The overall, 30 mg, and 60 mg analyses identified 25, 15, and 35 FDR-significant features, respectively; none was significant in the exploratory 15 mg group. Twelve features were shared across the primary analyses and increased at week 12; they comprised mainly acylcarnitines, together with docosadienoic acid and DG (21:0/16:0/0:0), and four lacked HMDB identifiers. The corresponding FDR panels yielded participant-grouped out-of-fold AUCs of 0.918 (95% CI 0.890–0.963), 0.786 (0.701–0.890), and 0.891 (0.835–0.950) for discriminating baseline from week 12, respectively. Pathway mapping identified no multi-compound enriched pathways (q < 0.05). Observed annotations represented single-metabolite signals—most notably DG (21:0/16:0/0:0) (HMDB0094301) and octanoylcarnitine (HMDB0000791)—rather than coordinated, pathway-level alterations. **Conclusions:** Paired analyses identified consistent longitudinal plasma lipidomic changes in patients receiving edoxaban. The 12 shared features provide focused candidates for targeted validation. Controlled studies are needed to determine whether these changes are specific to edoxaban and to establish their biological and clinical relevance.

## 1. Introduction

Atrial fibrillation (AF) is the most common sustained cardiac arrhythmia and is associated with substantial risks of ischemic stroke, systemic embolism, heart failure, and death [1]. Direct oral anticoagulants are recommended for stroke prevention in most eligible patients with nonvalvular AF because they provide predictable anticoagulation and favorable efficacy and safety compared with vitamin K antagonists [1,2]. Edoxaban is a direct factor Xa inhibitor whose efficacy and safety were established in the ENGAGE AF-TIMI 48 trial; its approved dosing incorporates renal function, body weight, and selected P-glycoprotein inhibitors [2,3,4].

Circulating lipids may reflect metabolic processes relevant to AF, including membrane remodeling, energy-substrate handling, and sphingolipid signaling. Population-based lipidomic and metabolomic studies have reported associations involving phospholipids, sphingolipids, and fatty acids, but findings differ by molecular species, cohort, platform, and statistical correction [5,6,7,8,9,10,11,12]. Most studies have compared participants with and without AF or examined incident AF. Pharmacometabolomic evidence during oral factor Xa inhibition is limited; one study assessed plasma metabolites 3 h after rivaroxaban dosing, a substantially different biological window from longer-term predose sampling [13].

The present study was a prespecified longitudinal lipidomic substudy of a prospective multicenter edoxaban pharmacokinetic study in Chinese patients with nonvalvular AF [4]. We aimed to characterize paired plasma lipidomic changes between baseline and week 12, identify features shared across the overall cohort and the 30 mg and 60 mg groups, evaluate the internal ability of FDR-significant panels to distinguish the two sampling time points, and explore HMDB-SMPDB pathway enrichment. The analyses were designed to identify consistent longitudinal lipidomic patterns and prioritize candidate features and pathways for targeted validation.

## 2. Materials and Methods

### 2.1. Study Design and Participants

This prespecified longitudinal lipidomic substudy was embedded in a prospective, multicenter, open-label, 12-week pharmacokinetic study of edoxaban in Chinese patients with nonvalvular AF. Participants were enrolled at Fuwai Hospital, Chinese Academy of Medical Sciences, and Aerospace Central Hospital. The parent study established a planned enrollment target of 120 participants, among whom 104 were ultimately enrolled and initiated edoxaban treatment. The parent population pharmacokinetic analysis has been reported separately [4], and the trial was registered at ClinicalTrials.gov (NCT05320627). The protocol received central ethics approval from the Ethics Committee of Fuwai Hospital, Chinese Academy of Medical Sciences (No. 2021-1600). All participants provided written informed consent, and the study was conducted in accordance with the Declaration of Helsinki and Good Clinical Practice guidelines.

Eligible participants were adults aged at least 20 years with nonvalvular AF requiring anticoagulation for at least 3 months, creatinine clearance (CrCl) of at least 15 mL/min calculated using the Cockcroft–Gault equation, and written informed consent. Principal exclusions were valvular AF or previous valve replacement, dialysis or CrCl below 15 mL/min, clinically important anemia or thrombocytopenia, active or recent major bleeding, antiplatelet therapy, contraindication to edoxaban, uncontrolled hypertension, clinically important hepatic injury, pregnancy, recent edoxaban exposure, participation in another clinical trial, or inability to complete protocol follow-up. The lipidomic analysis was restricted to participants with paired fasting predose plasma samples available at baseline (W0) and week 12 (W12) (Supplementary Figure S1).

### 2.2. Protocol-Defined Dose Allocation

Edoxaban dose allocation was prespecified in the parent-study protocol and was not randomized [4]. Participants with normal or mild renal impairment (CrCl ≥ 50 mL/min) and no dose-reduction criterion received 60 mg once daily. Participants with at least one of the following received 30 mg once daily: CrCl 15 to <50 mL/min, body weight ≤ 60 kg, or use of a protocol-defined P-glycoprotein inhibitor. Participants aged ≥ 80 years who also had at least one protocol-listed risk factor—including high bleeding risk, body weight ≤ 45 kg, CrCl 15 to <30 mL/min, or chronic nonsteroidal anti-inflammatory drug use—received 15 mg once daily.

### 2.3. Sample Collection and Processing

Fasting predose venous blood was collected into EDTA anticoagulant tubes at W0 and W12. The samples were processed within 2 h, centrifuged at 3000× *g* for 10 min at 4 °C, aliquoted, and stored at −80 °C until analysis. The parent study collected multiomic samples at W0, week 4, and W12; the present lipidomic substudy used the paired W0 and W12 samples specified in the lipidomic analysis plan.

### 2.4. Widely Targeted Lipidomics Analysis

Lipidomic analysis was performed by Metware Biotechnology Co., Ltd. (Wuhan, China). After thawing on ice, 50 µL plasma was mixed with 1 mL methyl tert-butyl ether/methanol (3:1, *v*/*v*) containing an internal-standard coeffic-standard mixture. The samples were vortexed for 15 min, mixed with 200 µL ultrapure water, vortexed for 1 min, and centrifuged at 12,000 rpm for 10 min at 4 °C. A 200 µL aliquot of the upper organic phase was vacuum-dried and reconstituted in 200 µL acetonitrile/isopropanol (1:1, *v*/*v*) [14].

Chromatographic separation used an ExionLC AD UPLC system (SCIEX, Framingham, MA, USA) coupled to a SCIEX QTRAP 6500+ mass spectrometer (SCIEX, Framingham, MA, USA). An Accucore C30 column (2.1 × 100 mm, 2.6 µm; Thermo Fisher Scientific, Waltham, MA, USA) was maintained at 45 °C, with a flow rate of 0.35 mL/min and injection volume of 2 µL. Mobile phase A was acetonitrile/water (60:40, *v*/*v*) and mobile phase B was acetonitrile/isopropanol (10:90, *v*/*v*); both contained 0.1% formic acid and 10 mmol/L ammonium formate. The A: B gradient was 80:20 at 0 min, 70:30 at 2 min, 40:60 at 4 min, 15:85 at 9 min, 10:90 at 14 min, 5:95 from 15.5 to 17.3 min, and 80:20 from 17.5 to 20 min.

Data were acquired in positive- and negative-ion electrospray modes using predefined multiple-reaction-monitoring (MRM) transitions [15]. Source settings were 500 °C, +5500 V in positive mode and −4500 V in negative mode, with gas 1, gas 2, and curtain gas set to 45, 55, and 35 psi, respectively. Analyst 1.6.3 controlled data acquisition, and MultiQuant was used for peak integration. Lipid features were annotated against the in-house Metware Database using retention time and precursor/product-ion transitions. The values used for statistical analysis were automatically integrated relative peak areas and represent relative abundance rather than absolute concentration. An internal-standard mixture was added during extraction and monitored for analytical stability, but internal standards were not used for feature-wise normalization. No sample-volume, total-ion-current, quality-control (QC)-based drift correction, or batch correction was applied. Annotations not confirmed with authentic standards were treated as putative, consistent with metabolomics reporting standards [16].

Pooled quality-control samples were prepared by combining equal volumes of study samples. Sixteen pooled-QC injections were acquired in a continuous sequence, as follows: a pooled QC was injected before each of 14 blocks of 10 study samples and before a final block of 8 study samples, and the run ended with a closing pooled-QC injection (148 study samples in total). Sample injection order was not randomized; samples were acquired in sample-number order, and paired baseline and week-12 samples were not injected adjacently. Analytical stability was evaluated using internal-standard performance and overlapped pooled-QC total-ion-chromatogram (TIC) profiles. Internal-standard channels were required to meet the vendor stability criterion of CV ≤ 15% [17]. Eight internal-standard channels were monitored in pooled-QC samples (Supplementary Table S1).

### 2.5. Data Preprocessing

During laboratory preprocessing, missing values in the initial widely targeted panel were imputed with one-fifth of the minimum nonmissing value for the corresponding feature. Features with pooled-QC CV < 30% were retained, yielding a vendor-delivered analysis matrix of 1099 lipid features, consistent with the QC acceptance criterion reported for plasma pseudotargeted metabolomics [18]. Relative peak areas were transformed as log_2_(x + 1) before statistical analysis [19,20]. The same preprocessing was applied to the overall cohort and each dose-group analysis. A preprocessing and paired-sample summary is provided in Supplementary Table S2.

### 2.6. Differential Lipid Analysis

Two-sided paired *t*-tests compared transformed W0 and W12 abundances within participants. For each comparison, log_2_FC was defined as the mean log_2_(W12 + 1) abundance minus the mean log_2_(W0 + 1) abundance. The 95% confidence interval for log_2_FC was a paired t interval calculated from the within-participant differences d_i_ = log_2_(W12 + 1)_i_ − log_2_(W0 + 1)_i_. Benjamini–Hochberg correction was applied separately to all 1099 tests in each comparison [21]. A feature was considered FDR-significant when both |log_2_FC| > 0.58 and q < 0.05. Nominal results meeting |log_2_FC| > 0.58 and *p* < 0.05 were retained only for descriptive supplementary reporting. Only FDR-significant features were used for shared-feature, machine-learning, and pathway analyses. The overall cohort, 30 mg group, and 60 mg group were primary analyses; the 15 mg group was exploratory because only three participants had paired samples.

### 2.7. Candidate Lipid Panels and Machine-Learning Analysis

For each primary comparison, the candidate panel comprised all FDR-significant lipids. The primary shared set was defined as the intersection of the overall, 30 mg, and 60 mg FDR-significant sets. The exploratory 15 mg group was excluded from this definition.

Random forest classifiers were used for the overall cohort and 60 mg group. In the 30 mg group, a LASSO-based feature-compression procedure was followed by logistic regression. Five-fold GroupKFold cross-validation used participant ID as the grouping variable, so paired W0 and W12 samples from the same participant remained in the same fold. Scaling parameters were fitted using training-fold data only. Receiver-operating-characteristic curves and areas under the curve (AUCs) were calculated from pooled out-of-fold predicted probabilities using scikit-learn [22]. Patient-level bootstrap 95% confidence intervals were calculated for the FDR-panel AUCs by resampling participants with replacement while keeping paired W0/W12 samples together; the AUC point estimates remained those from the original participant-grouped cross-validation. For descriptive comparison, models using all retained lipid features were evaluated with the same participant-grouped procedure. Machine-learning importance was used only to rank lipids within the FDR-defined panels and did not redefine statistical significance.

### 2.8. Pathway Enrichment Analysis

Human Metabolome Database (HMDB) identifiers were mapped to Small Molecule Pathway Database (SMPDB) pathways using the HMDB XML resource [23,24]. One-sided Fisher exact tests assessed over-representation, followed by Benjamini–Hochberg correction. The study-specific background comprised 331 unique detected HMDB identifiers that mapped to at least one SMPDB pathway. Separate enrichment analyses were performed for the overall cohort, 30 mg group, 60 mg group, and primary shared set using their FDR-significant HMDB identifiers against the study-specific background (*n*= 331 pathway-mapped study HMDB IDs; Supplementary Figure S2 and Supplementary Table S3A,B). The exploratory 15 mg group had no FDR-significant input and was not subjected to primary pathway enrichment. To avoid overinterpreting pathway-level inferences from overlapping species-specific SMPDB annotations, FDR-significant mapped entries were inspected for compound coverage. Entries driven by a single HMDB identifier (k = 1) were interpreted as isolated single-metabolite annotation signals rather than as evidence of coordinated pathway-level alteration.

### 2.9. Statistical Analysis

Statistical analyses were performed in Python 3.12.7. All hypothesis tests were two-sided except the prespecified one-sided over-representation tests. Continuous lipidomic effect estimates are reported as paired mean log_2_FC with 95% confidence intervals, and multiple-testing-adjusted values are reported as q values. Participant and sample counts are reported separately to distinguish paired participants from individual plasma samples.

## 3. Results

### 3.1. Participant Disposition, Baseline Characteristics, and Analytical QC

The parent study planned to enroll 120 participants, with 40 participants in each dose group. A total of 104 participants were enrolled and initiated edoxaban treatment, including 4, 31, and 69 participants in the 15 mg, 30 mg, and 60 mg groups, respectively (Supplementary Figure S1). Paired W0 and W12 lipidomic samples were available for 74 participants (15 mg, *n* = 3; 30 mg, *n* = 23; and 60 mg, *n* = 48), yielding 148 plasma samples. The remaining 30 treated participants were not included in the paired lipidomic analysis because paired W0/W12 samples were unavailable (Supplementary Figure S1). Baseline characteristics of the paired analytical cohort are summarized in Table 1.

Analytical reliability of the lipidomic measurements was supported by laboratory QC metrics. Internal-standard CVs in pooled-QC samples were approximately 2.0–5.2%, indicating good analytical stability and run-to-run reproducibility. Overlapped pooled-QC TIC profiles in positive- and negative-ion modes showed close overlay across repeated injections, indicating technical reproducibility of extraction and detection (Supplementary Figure S3).

### 3.2. Longitudinal Lipidomic Changes Between Baseline and Week 12

#### 3.2.1. FDR-Significant Differential Lipids

**Figure 1:**
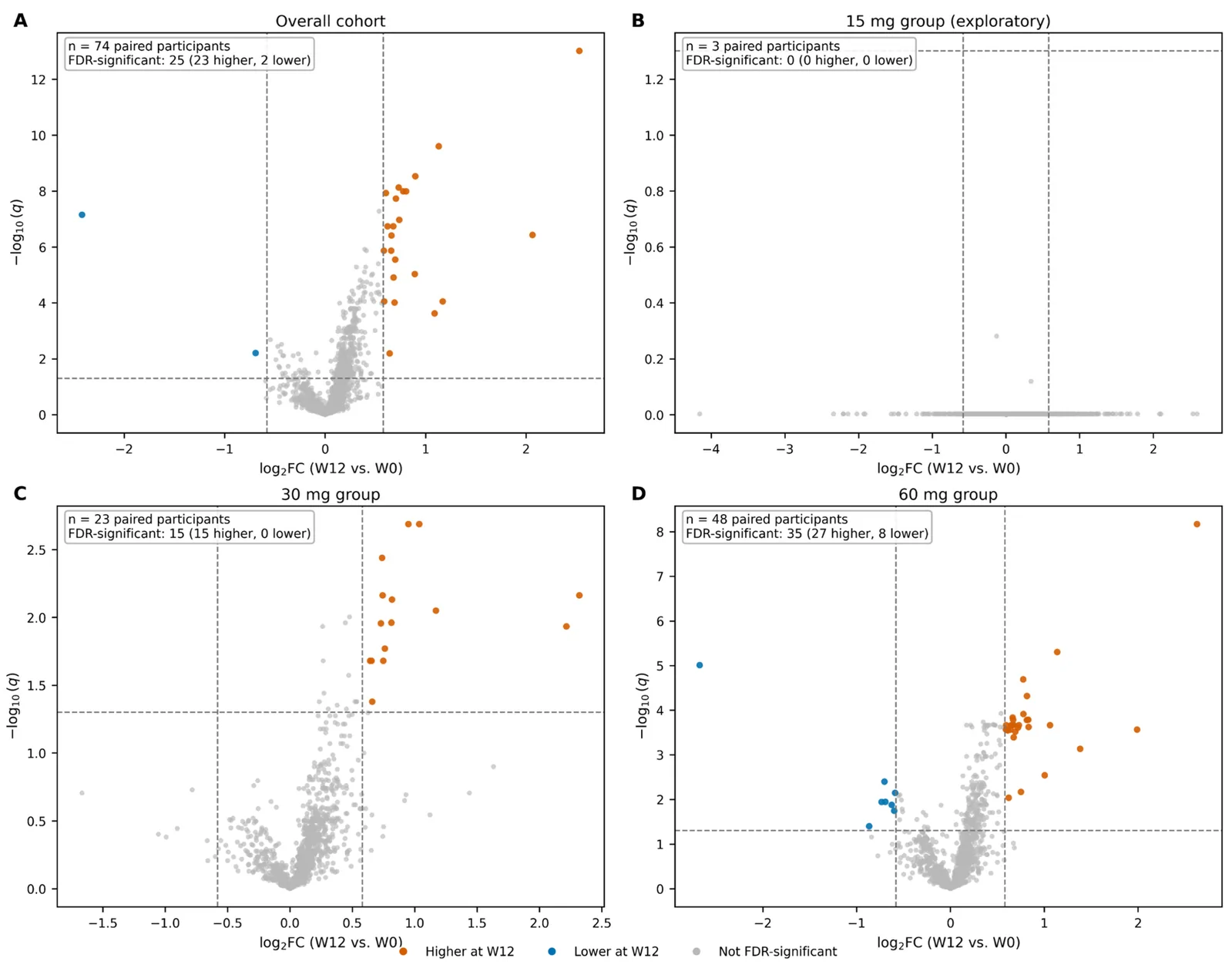
FDR-significant longitudinal lipidomic changes between baseline and week 12. Volcano plots show paired comparisons in (**A**) the overall cohort, (**B**) the exploratory 15 mg group, (**C**) the 30 mg group, and (**D**) the 60 mg group. Each point represents one of 1099 lipid features. The *x*-axis is log_2_FC (W12 versus W0), and the *y*-axis is −log_10_(q). Vertical dashed lines indicate log_2_FC = −0.58 and 0.58; the horizontal dashed line indicates q = 0.05. Higher-at-W12, lower-at-W12, and nonsignificant features are distinguished by color. Panel summaries, expressed as paired participants, are as follows: (**A**) *n* = 74, 25 FDR-significant features (23 higher, 2 lower); (**B**) *n* = 3, no FDR-significant feature; (**C**) *n* = 23, 15 FDR-significant features (all higher); and (**D**) *n* = 48, 35 FDR-significant features (27 higher, 8 lower).

Using |log_2_FC| > 0.58 and q < 0.05, 25 features were FDR-significant in the overall cohort; 23 had higher and 2 had lower relative abundance at W12. The 30 mg analysis identified 15 FDR-significant features, all higher at W12. The 60 mg analysis identified 35 features, of which 27 were higher and 8 lower at W12 (Table 2; Figure 1). Paired log_2_FC values with 95% CIs are reported in Supplementary Table S4.

No feature met the FDR criteria in the exploratory 15 mg analysis. Twenty-one features met the nominal criteria of |log_2_FC| > 0.58 and *p* < 0.05, but none remained significant after multiple-testing correction (Supplementary Table S4). Consistent with its exploratory designation, the 15 mg subgroup was not included in the primary shared-feature, machine-learning, or pathway analyses.

#### 3.2.2. Shared FDR-Significant Lipids Across the Primary Analyses

**Figure 2:**
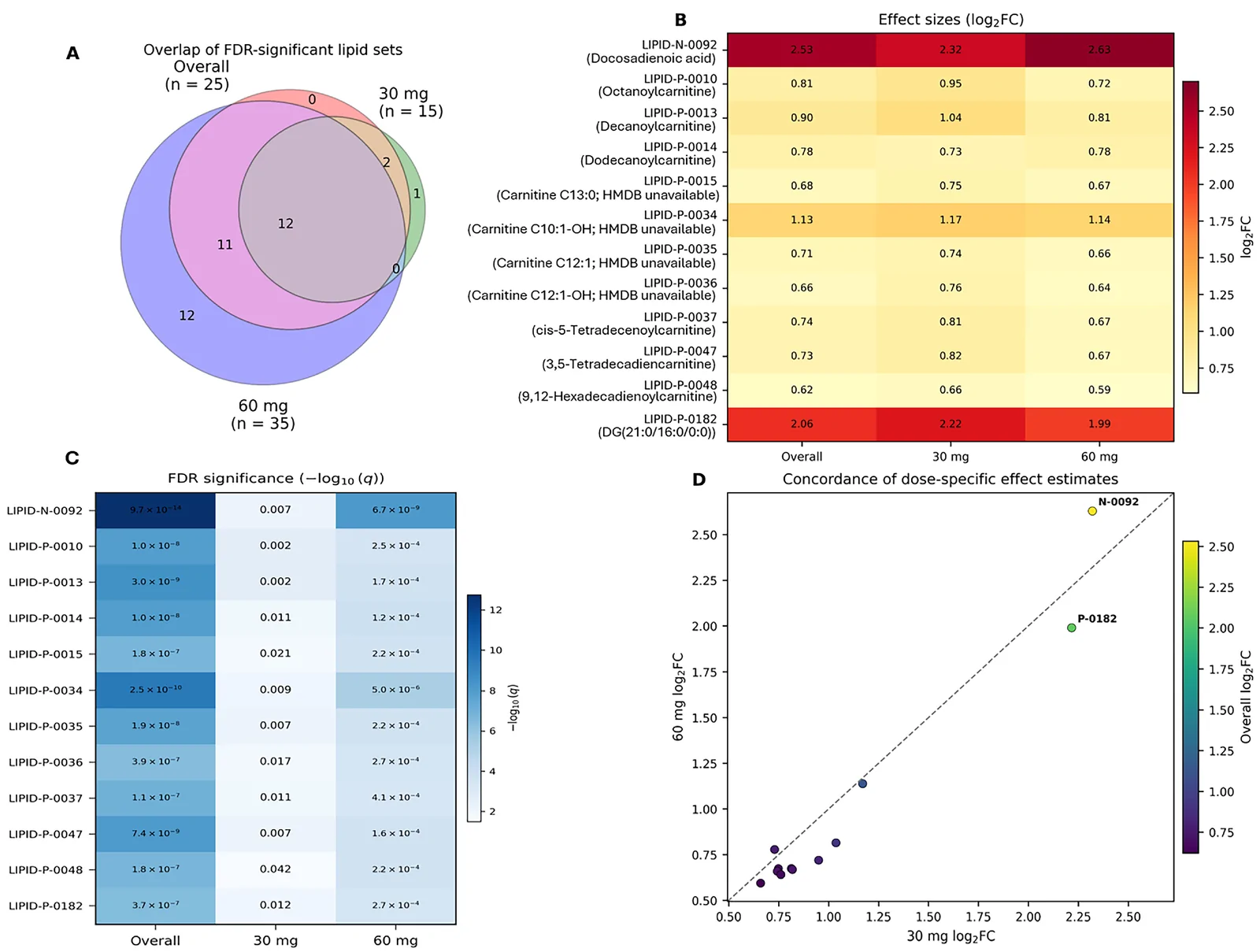
Shared FDR-significant lipid alterations across the primary longitudinal analyses. (**A**) Venn diagram showing overlap among the overall cohort (25 features), 30 mg group (15 features), and 60 mg group (35 features), with 12 features in the three-way intersection. The exploratory 15 mg analysis was not used to define the shared set. (**B**) Heatmap of log_2_FC values for the 12 shared features across the three primary analyses. (**C**) Heatmap of −log_10_(q) values. (**D**) Concordance of dose-specific log_2_FC values in the 30 mg and 60 mg analyses; point color represents the overall-cohort log_2_FC. LIPID-N-0092 and LIPID-P-0182 are labeled as examples with large, concordant changes. All 12 features were higher at W12 and met |log_2_FC| > 0.58 and q < 0.05 in each primary analysis.

The intersection of the overall, 30 mg, and 60 mg FDR-significant sets contained 12 features, all of which had higher relative abundance at W12 in each primary analysis (Figure 2). Eight features had HMDB identifiers and were named using HMDB common names, as follows: octanoylcarnitine, decanoylcarnitine, dodecanoylcarnitine, cis-5-tetradecenoylcarnitine, 3,5-tetradecadiencarnitine, 9,12-hexadecadienoylcarnitine, docosadienoic acid, and DG(21:0/16:0/0:0). The remaining four features had no HMDB identifier and were annotated by the vendor as acylcarnitines. Vendor compound names, Class II subclasses, and HMDB identifiers are reported in Supplementary Table S5 (e.g., LIPID-N-0092 vendor Class II: free fatty acid; HMDB0251556: docosadienoic acid). LIPID-N-0092 (HMDB0251556; docosadienoic acid) had log_2_FC values of 2.53, 2.32, and 2.63 in the overall, 30 mg, and 60 mg analyses, respectively. LIPID-P-0182 (HMDB0094301; DG(21:0/16:0/0:0)) had corresponding log_2_FC values of 2.06, 2.22, and 1.99. Vendor class and analytical metadata are provided in Supplementary Table S5. Paired log_2_FC values with 95% CIs, P, and q values for these 12 features are provided in Supplementary Table S6.

### 3.3. Discriminative Performance of FDR-Significant Lipid Panels

#### 3.3.1. Machine-Learning Ranking of Candidate Lipid Features

**Figure 3:**
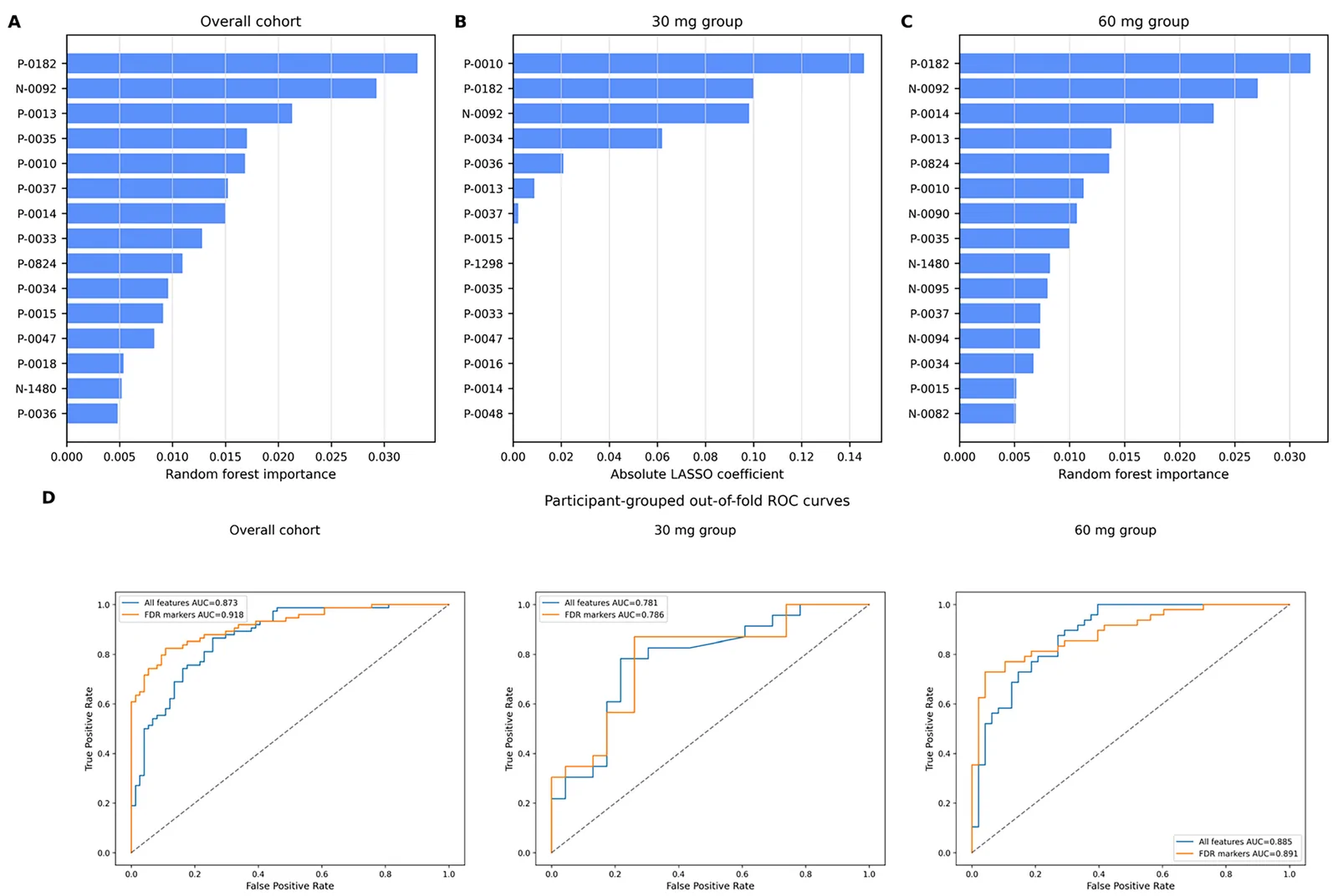
Machine-learning ranking and participant-grouped cross-validated discrimination of FDR-significant lipid panels. (**A**) Random forest importance ranking for the overall cohort. (**B**) Absolute LASSO coefficients for the 30 mg group; zero coefficients do not remove features from the prespecified 15-feature FDR panel. (**C**) Random forest importance ranking for the 60 mg group. Rankings were descriptive and did not redefine FDR significance. (**D**) Participant-grouped out-of-fold ROC curves for W0 versus W12 classification using all retained features and the corresponding FDR panels. FDR-panel AUCs were 0.918, 0.786, and 0.891 for the overall, 30 mg, and 60 mg analyses, respectively. These curves represent internal time-point discrimination and not clinical diagnostic or treatment-response performance.

The candidate panels contained 25, 15, and 35 FDR-significant features in the overall, 30 mg, and 60 mg analyses, respectively. Machine-learning algorithms ranked features within these predefined panels and did not introduce non-FDR-significant features. The highest-ranked features were LIPID-P-0182, LIPID-N-0092, and LIPID-P-0013 in the overall cohort; LIPID-P-0010, LIPID-P-0182, and LIPID-N-0092 in the 30 mg group; and LIPID-P-0182, LIPID-N-0092, and LIPID-P-0014 in the 60 mg group (Figure 3A–C; Supplementary Table S7A).

#### 3.3.2. Participant-Grouped Cross-Validated Discrimination Between W0 and W12

Using pooled out-of-fold probabilities from five-fold participant-grouped cross-validation, the FDR panels yielded AUCs of 0.918 (95% CI 0.890–0.963) in the overall cohort, 0.786 (0.701–0.890) in the 30 mg group, and 0.891 (0.835–0.950) in the 60 mg group (Table 3; Figure 3D). Models using all 1099 retained features yielded AUCs of 0.873, 0.781, and 0.885, respectively. Cross-validation settings and FDR-panel AUCs with 95% CIs are summarized in Supplementary Table S7B. Vendor platform annotations for all 1099 retained lipid features are provided in Supplementary Table S8. These analyses evaluated internal discrimination between W0 and W12 samples only and were not designed to predict treatment response, clinical outcomes, or dose-group membership. Because panel membership was defined before cross-validation and no independent validation cohort was available, the AUCs should be interpreted as exploratory internal estimates.

### 3.4. Pathway Mapping and Enrichment

Among FDR-significant lipids, 15 unique HMDB identifiers were available in the overall cohort, of which 3 mapped to at least one SMPDB pathway. The corresponding input/mapped counts were 9/2 in the 30 mg group, 24/8 in the 60 mg group, and 8/2 in the 12-feature shared set. All analyses used 331 pathway-mapped study HMDB identifiers as the background (Table 4; Supplementary Table S3A).

In the overall cohort, 31 FDR-significant SMPDB entries were observed. Of these, 29 entries related to de novo triacylglycerol biosynthesis (representative q ≈ 0.0097) were driven exclusively by LIPID-P-0182 (HMDB0094301; DG(21:0/16:0/0:0)), one mitochondrial short-chain fatty-acid β-oxidation annotation was driven by LIPID-P-0010 (HMDB0000791; octanoylcarnitine; q ≈ 0.018), and one additional triacylglycerol-related entry was driven by HMDB0005368 (q ≈ 0.018). These closely related, species-specific database entries constitute single-metabolite annotation clusters and should not be counted as independent pathways or as coordinated pathway regulation. The 30 mg analysis and the shared-feature analysis showed 29 TG-related entries (representative q ≈ 0.0063) plus the same β-oxidation annotation (q ≈ 0.012). In the 60 mg analysis, 30 FDR-significant SMPDB entries were observed (representative q ≈ 0.032), comprising TG-related single-metabolite annotations driven by HMDB0094301 and HMDB0043019; mitochondrial β-oxidation driven by HMDB0000791 did not reach FDR significance (q = 0.053). Complete mapping and enrichment results are shown in Supplementary Figure S2 and Supplementary Table S3A,B.

## 4. Discussion

### 4.1. Principal Findings

This longitudinal substudy identified distinct plasma lipidomic changes between W0 and W12 in Chinese patients with NVAF receiving edoxaban. Twenty-five, 15, and 35 lipid features met the FDR criteria in the overall cohort, 30 mg group, and 60 mg group, respectively, and 12 features were consistently shared across the three primary analyses with higher relative abundance at W12. These shared features were predominantly acylcarnitines, together with docosadienoic acid and DG (21:0/16:0/0:0); four features lacked HMDB identifiers. The FDR-defined panels also captured a consistent multivariate distinction between W0 and W12 in participant-grouped internal cross-validation.

### 4.2. Biological Interpretation of the Longitudinal Lipid Changes

The 12 shared features comprised six HMDB-annotated acylcarnitines, docosadienoic acid, DG (21:0/16:0/0:0), and four features without HMDB identifiers that were vendor-annotated as acylcarnitines. Vendor compound names, Class II subclasses, and HMDB identifiers are reported in Supplementary Table S5. Their concordant increase across the overall, 30 mg, and 60 mg analyses indicates a longitudinal plasma lipidomic signal carried mainly by acylcarnitines, together with a diacylglycerol and docosadienoic acid. Prior metabolomic studies have reported acylcarnitine perturbations in AF and related cardiovascular conditions, consistent with altered mitochondrial fatty-acid handling [8,9,10,11,12]. These features provide a candidate set for targeted quantification and structural confirmation.

Previous lipidomic and metabolomic studies support a broader role of circulating lipids in AF, although the molecular classes implicated have varied across cohorts. Prospective analyses from the Cardiovascular Health Study linked individual ceramide, sphingomyelin, and plasma phospholipid fatty-acid species with incident AF [5,6], and PREDIMED identified an AF-associated multilipid network enriched in phosphatidylcholine- and phosphatidylethanolamine-related species [7]. Findings from Framingham, ARIC, and other clinical or multi-omic cohorts further implicated lipid, fatty-acid, glycerophospholipid, purine, and intermediary metabolism [8,9,10,11,12]. Against this background, the present shared longitudinal set—dominated by acylcarnitines together with docosadienoic acid and DG (21:0/16:0/0:0)—does not recapitulate the phospholipid- and sphingolipid-centered signatures reported in those AF cohorts. It instead points to fatty-acid transport and oxidation-related acylcarnitines, a diacylglycerol, and docosadienoic acid as candidate contributors to the observed temporal change, pending targeted structural confirmation.

Pharmacometabolomic evidence during oral factor Xa inhibition remains limited. Zhao et al. reported plasma metabolic changes 3 h after rivaroxaban administration in patients with NVAF [13]. In contrast, the present study used fasting predose samples collected at baseline and week 12, thereby capturing a longer-term longitudinal pattern rather than an immediate postdose response. The shared acylcarnitine, docosadienoic acid, and DG features therefore provide focused candidates for validation in controlled longitudinal studies, while their drug specificity and biological mechanisms remain to be established.

### 4.3. Interpretation of the Machine-Learning and Pathway Findings

The participant-grouped machine-learning analyses complemented the univariate findings by showing that the FDR-significant panels captured coordinated multivariate differences between W0 and W12. Across the primary analyses, these focused panels achieved discrimination comparable to or slightly better than models containing all 1099 retained features, suggesting that the longitudinal signal was concentrated within a relatively limited feature set. With patient-level bootstrap 95% confidence intervals now reported for the FDR-panel AUCs (Table 3), the overall and 60 mg estimates remain supportive of internal discrimination, whereas the wider interval in the 30 mg group (*n* = 23) indicates substantially reduced precision. Accordingly, we regard the reported AUCs as exploratory internal estimates of sampling-time-point separation rather than evidence of stable, clinically actionable prediction; panel definition was not fully nested, and no external validation cohort was available. The recurrent high ranking of LIPID-P-0182 and LIPID-N-0092, together with their consistent increases across the primary comparisons, further prioritized these features and the broader 12-feature shared set for targeted quantification and structural confirmation. This panel-level pattern is consistent with previous AF studies in which multimetabolite or lipid-network approaches provided information beyond individual molecular associations [7,10,11,12].

Pathway mapping provided preliminary biological context for these longitudinal lipid changes. Previous human atrial-tissue studies have reported alterations in energy-substrate utilization and mitochondrial processes in AF [25,26]. However, observed SMPDB annotations—specifically for de novo triacylglycerol biosynthesis and short-chain fatty-acid β-oxidation—were centered entirely on single mapped metabolites, diacylglycerol (HMDB0094301), and octanoylcarnitine (HMDB0000791), respectively. Because these signals stem from isolated HMDB identifiers rather than coordinated, multi-compound pathway alterations (typically k = 1; enrichment ratio = 1), they should be interpreted as hypothesis-generating indicators focusing on specific lipid species rather than direct evidence of pathway-level reprogramming. In the 60 mg analysis, 30 FDR-significant SMPDB entries were likewise driven by single triglyceride/diacylglycerol identifiers (HMDB0094301 and HMDB0043019), without multi-compound (k ≥ 2) pathway hits.

### 4.4. Strengths and Limitations

Strengths of this study include the prospective multicenter design, standardized fasting predose sampling, paired within-participant comparisons, FDR-controlled feature selection, participant-grouped cross-validation, and use of a study-specific pathway background. Public deposition of the lipidomic dataset and provision of complete statistical and pathway outputs further enhance transparency and reproducibility.

Several limitations should be considered. Without an untreated or active-anticoagulant comparator, longitudinal changes cannot be attributed specifically to edoxaban. Dose allocation was nonrandom and clinically determined, yielding unequal and potentially confounded dose groups, particularly the exploratory 15 mg subgroup. Sample injection order was not randomized, and paired baseline and week-12 samples were not injected adjacently. Comorbidities and concomitant medications were not incorporated as covariates. Relative peak areas and partly putative annotations limit quantitative and structural inference. Pathway and machine-learning findings remain exploratory and require validation in independent, controlled cohorts.

### 4.5. Implications

The shared acylcarnitine, docosadienoic acid, and DG features provide a focused set of candidates for controlled longitudinal validation. Future studies should include untreated or active-comparator groups, larger and more balanced cohorts, serial sampling, absolute targeted quantification, detailed medication and dietary assessment, pharmacokinetic and pharmacodynamic measurements, clinical outcomes, and external model validation. Such validation will be necessary before the panels can be evaluated for therapeutic monitoring, risk prediction, or dose selection.

## 5. Conclusions

This study delineated a coherent longitudinal plasma lipidomic pattern between baseline and week 12 in Chinese patients with nonvalvular AF receiving edoxaban. Twelve shared FDR-significant features—predominantly acylcarnitines, together with docosadienoic acid and DG (21:0/16:0/0:0)—increased consistently at week 12 and distinguished the two sampling time points in participant-grouped internal validation. These findings define a focused candidate set for targeted quantification, structural confirmation, and mechanistic investigation, expand current understanding of lipidomic dynamics in patients with NVAF receiving anticoagulation, and provide a foundation for controlled longitudinal studies integrating serial sampling and pharmacokinetic/pharmacodynamic measurements to clarify drug specificity, biological significance, and translational potential.

## Figures and Tables

**Table 1:** Baseline demographic and clinical characteristics of the paired lipidomic analytical cohort.

Characteristic	15 mg (*n* = 3)	30 mg (*n* = 23)	60 mg (*n* = 48)	Overall (*n* = 74)
Age, years				
Mean ± SD	81.7 ± 2.1	64.0 ± 10.1	61.6 ± 9.6	63.1 ± 10.3
Median [min, max]	81 [80, 84]	65 [46, 80]	65 [38, 78]	65 [38, 84]
Male sex, ***n*** (%)	1 (33.3)	17 (73.9)	11 (22.9)	29 (39.2)
CrCl, mL/min				
Mean ± SD	38.7 ± 28.2	75.4 ± 25.8	97.1 ± 28.8	88 ± 31
Median [min, max]	23.87 [23.27, 68.94]	71.75 [35.05, 131.17]	89.9 [50.95, 182.6]	83.345 [23.27, 182.6]
Creatinine, µmol/L				
Mean ± SD	150.6 ± 88.9	69.2 ± 29.5	75.4 ± 14.3	76.5 ± 29.2
Median [min, max]	178.9 [51, 222]	63.2 [40.65, 190.8]	72.2 [53.9, 121.7]	69.45 [40.65, 222]
Weight, kg				
Mean ± SD	63.6 ± 6.6	57.4 ± 8.6	75.7 ± 10.4	69.5 ± 12.9
Median [min, max]	62 [57, 70]	58 [45, 81]	75 [60, 108]	69.5 [45, 108]
Concomitant P-gp inhibitors, ***n*** (%)	0 (0)	4 (17.4)	1 (2.1)	5 (6.8)
Concomitant amiodarone, ***n*** (%)	0 (0)	7 (30.4)	18 (37.5)	25 (33.8)

Note: Continuous variables are presented as mean ± SD or median [min, max]; categorical variables are presented as *n* (%). Because dose allocation was nonrandomized and protocol-defined based on clinical criteria, Table 1 is descriptive, and no formal inferential statistical tests are reported. CrCl, creatinine clearance; NSAID, nonsteroidal anti-inflammatory drug; P-gp, P-glycoprotein; SD, standard deviation.

**Table 2:** Summary of FDR-significant longitudinal lipidomic changes between baseline and week 12.

Analysis Set	Paired Participants	Lipids Tested	FDR-Significant Lipids	Higher at W12	Lower at W12	Status
Overall cohort	74	1099	25	23	2	Primary
15 mg group	3	1099	0	0	0	Exploratory
30 mg group	23	1099	15	15	0	Primary
60 mg group	48	1099	35	27	8	Primary

Note: FDR-significant lipids met both |log_2_FC| > 0.58 and Benjamini–Hochberg-adjusted q < 0.05. Direction is relative to W0. FC, fold change; FDR, false discovery rate; W0, baseline; W12, week 12.

**Table 3:** Participant-grouped cross-validated discrimination between baseline and week-12 samples.

Analysis Set	Participants	Samples	Algorithm	FDR Panel Size	AUC, All Features	AUC, FDR Panel (95% CI)
Overall cohort	74	148	Random forest	25	0.873	0.918 (0.890–0.963)
30 mg group	23	46	LASSO-based compression + logistic regression	15	0.781	0.786 (0.701–0.890)
60 mg group	48	96	Random forest	35	0.885	0.891 (0.835–0.950)

Note: AUCs were calculated from pooled out-of-fold predictions generated using five-fold GroupKFold cross-validation with participant ID as the grouping variable. Paired W0 and W12 samples remained in the same fold. FDR panels were defined in the full corresponding analysis sets before cross-validation; estimates are internal and may be optimistic. AUC, area under the receiver-operating-characteristic curve; FDR, false discovery rate; LASSO, least absolute shrinkage and selection operator.

**Table 4:** Summary of HMDB–SMPDB pathway annotation (single-metabolite–driven enrichment entries).

Analysis Set	FDR Lipids	HMDB IDs, Input/Mapped	Significant SMPDB Entries	Markers per Entry (k)	Multi-Metabolite Hits (k ≥ 2)
Overall cohort	25	15/3	31	1	0
15 mg group	0	0/0	Not analyzed	-	-
30 mg group	15	9/2	30	1	0
60 mg group	35	24/8	30	-	0
Shared 12-feature set	12	8/2	30	1	0

Note: Pathway enrichment analysis was performed using one-sided Fisher’s exact tests with Benjamini–Hochberg FDR correction (q < 0.05). After removing species-redundant and single-metabolite-driven database artifacts, no pathways met the criteria for multi-compound pathway-level enrichment. Reported entries represent database annotations driven exclusively by single compound identifiers and are presented for exploratory mapping rather than evidence of coordinated biological pathway regulation.

## Data Availability

The data reported in this paper have been deposited in the OMIX, China National Center for Bioinformation/Beijing Institute of Genomics, Chinese Academy of Sciences (https://ngdc.cncb.ac.cn/omix; accession no. OMIX018783), under the project title “Plasma lipidomics of Chinese patients with nonvalvular atrial fibrillation before and after 12-week edoxaban treatment”. Complete downstream differential-analysis, machine-learning, and pathway-enrichment outputs are provided in the Supplementary Materials.

## Supplementary Materials

The following supporting information can be downloaded at: https://www.mdpi.com/article/10.3390/metabo16090672/s1, Figure S1: Flow of participant selection for the paired lipidomic cohort; Figure S2: Summary of HMDB–SMPDB pathway enrichment using FDR-significant lipid sets; Figure S3: Overlapped TIC profiles of pooled-QC samples; Table S1: Characteristics and reproducibility of lipid internal standards in quality control (QC) samples; Table S2: Preprocessing and paired-sample summary; Table S3A: HMDB-to-SMPDB mapping statistics for the pathway-enrichment analyses; Table S3B: Complete HMDB–SMPDB pathway-enrichment results; Table S4: Complete paired differential-lipid results for all 1099 lipid features in the overall cohort and dose-specific analyses; Table S5: Vendor platform annotations for the 12 shared FDR-significant lipid features; Table S6: Detailed annotations and longitudinal statistics for the 12 shared FDR-significant features; Table S7: (A) Complete machine-learning feature rankings of the FDR-significant lipid panels; (B) Patient-grouped cross-validation performance summary; Table S8: Vendor platform annotations for all 1099 retained lipid features.

## Abbreviations

AF	Atrial fibrillation
AUC	Area under the curve
CAD	Collision-activated dissociation
CE	Collision energy
CrCl	Creatinine clearance
CUR	Curtain gas
CV	coefficients of variation (CVs)
DP	Declustering potential
ESI	Electrospray ionization
FC	Fold change
FDR	False discovery rate
FXa	Factor Xa
GS1	Gas 1
GS2	Gas 2
HMDB	Human Metabolome Database
LASSO	Least Absolute Shrinkage and Selection Operator
MRM	Multiple reaction monitoring
MWDB	Metware Database
NSAIDs	Non-steroidal anti-inflammatory drugs
NVAF	Nonvalvular atrial fibrillation
QC	Quality control
ROC	Receiver operating characteristic
RSD	Relative standard deviation
RT	Retention time
ULN	Upper limit of normal
UPLC-MS/MS	Ultra-performance liquid chromatography–tandem mass spectrometry
