# Supplementary material for: Longitudinal Plasma Lipidomic Changes in Chinese Patients with Nonvalvular Atrial Fibrillation During 12 Weeks of Edoxaban Treatment
Source: Metabolites. 2026 Sep 11;16(9):672. doi: 10.3390/metabo16090672 (PMC13608962; doi:10.3390/metabo16090672)
Supplement: Supplementary file 1 [file metabolites-16-00672-s001.zip › metabolites-4390008-Supplementary Figures.pdf]

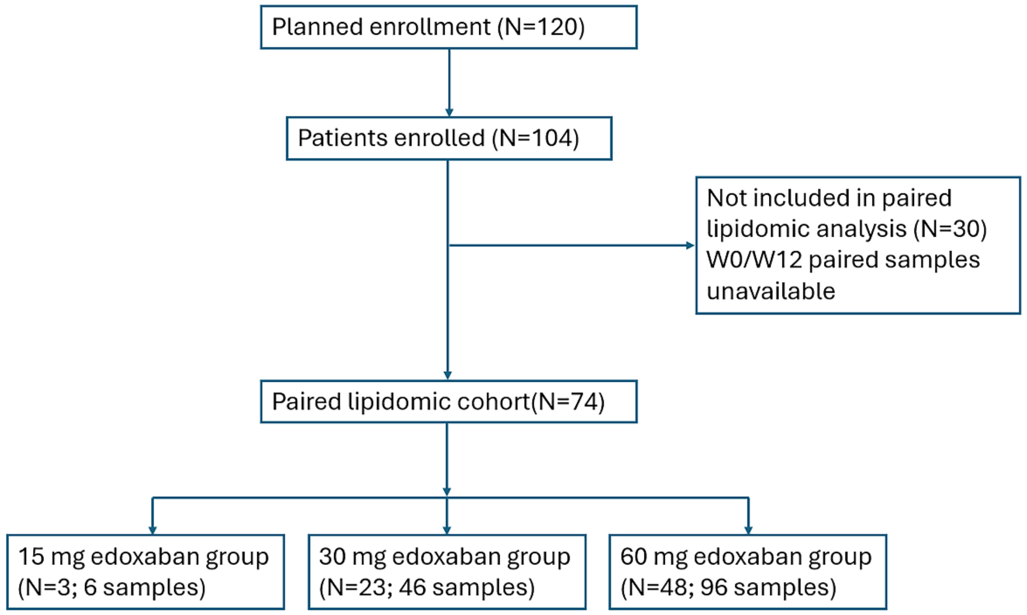

Supplementary Figure S1. Flow of participant selection for the paired lipidomic cohort (planned enrollment, N = 120; enrolled, N = 104). Thirty treated patients without paired W0/W12 samples were excluded from the paired analysis. The analytical cohort comprised 74 patients with paired samples (148 plasma samples): 15 mg, n = 3 (6 samples); 30 mg, n = 23 (46 samples); 60 mg, n = 48 (96 samples). W0, baseline; W12, week 12.

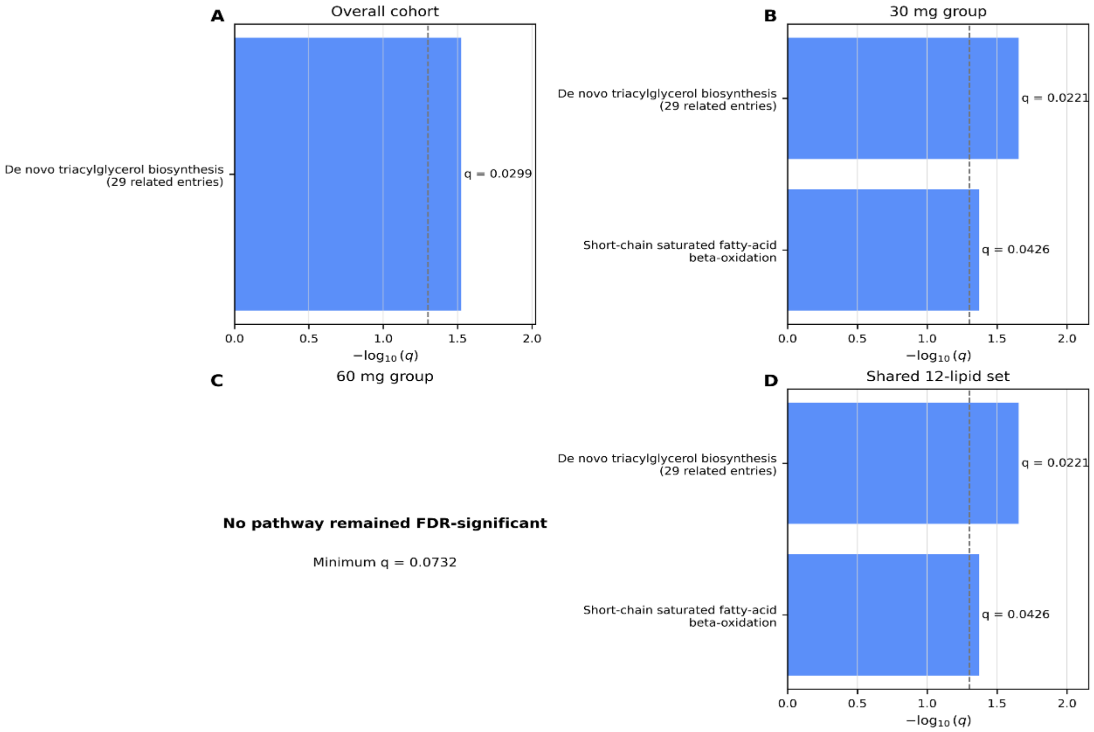

Supplementary Figure S2. Summary of HMDB–SMPDB pathway enrichment using FDR-significant lipid sets. Bars show $-\log_{10}(q)$ for pathways with $q < 0.05$ (dashed line) against a background of 331 pathway-mapped HMDB identifiers. (A) Overall cohort. (B) 30 mg group. (C) 60 mg group (no FDR-significant pathway; minimum q = 0.0732). (D) Shared 12-feature set.

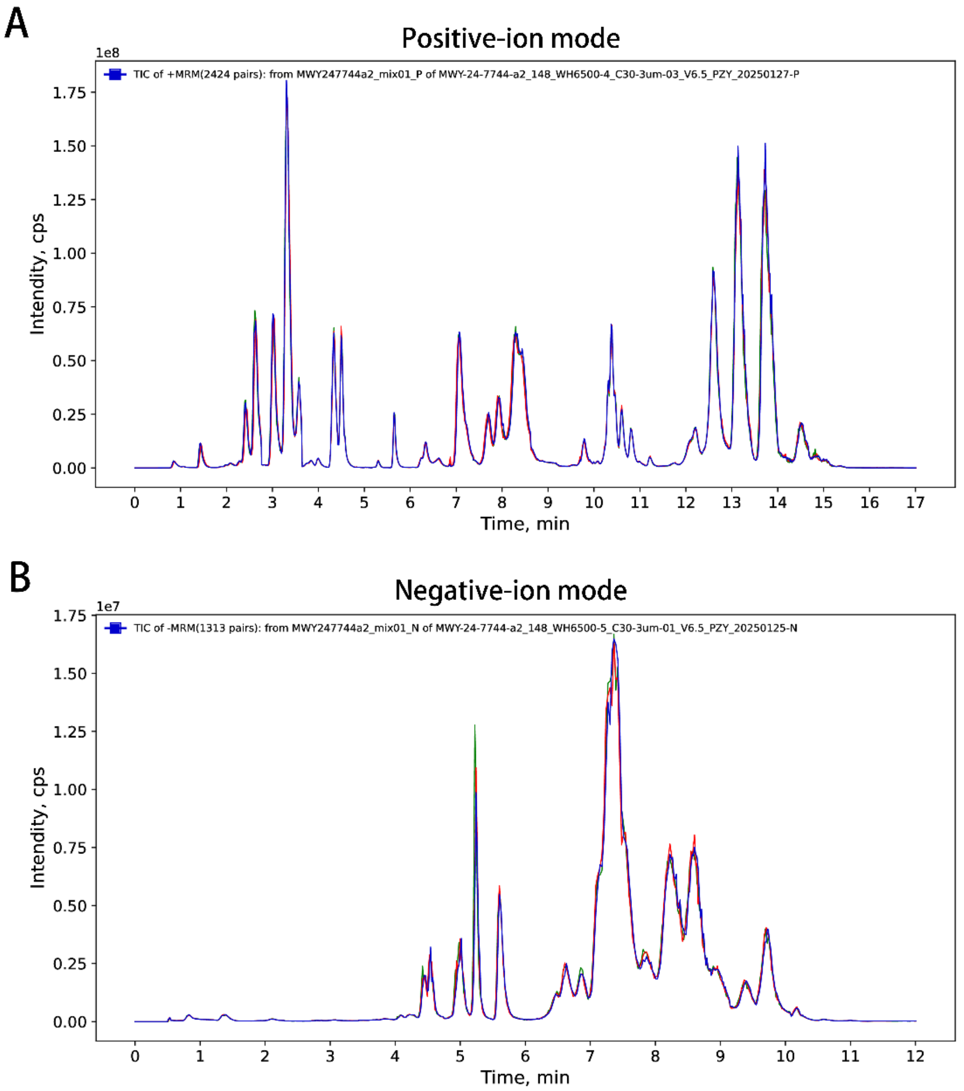

Supplementary Figure S3. Overlapped TIC profiles of pooled-QC samples. (A) Positive-ion mode. (B) Negative-ion mode. Close overlay of repeated QC injections indicates technical reproducibility.
